# Electrically switchable organo–inorganic hybrid for a white-light laser source

**DOI:** 10.1038/srep28363

**Published:** 2016-06-21

**Authors:** Jui-Chieh Huang, Yu-Cheng Hsiao, Yu-Ting Lin, Chia-Rong Lee, Wei Lee

**Affiliations:** 1 Institute of Photonic System, College of Photonics, National Chiao Tung University, Guiren Dist., Tainan 71150, Taiwan; 2 Institute of Imaging and Biomedical Photonics, College of Photonics, National Chiao Tung University, Guiren Dist., Tainan 71150, Taiwan; 3 Department of Photonics, National Cheng Kung University, Tainan 70101, Taiwan

## Abstract

We demonstrate a spectrally discrete white-light laser device based on a photonic bandgap hybrid, which is composed of a soft photonic crystal; i.e., a layer of dye-doped cholesteric liquid crystal (CLC), sandwiched between two imperfect but identical, inorganic multilayer photonic crystals. With a sole optical pump, a mono-, bi-, or tri-chromatic laser can be obtained and, through the soft photonic crystal regulated by an applied voltage, the hybrid possesses electrical tunability in laser wavelength. The three emitted spectral peaks originate from two bandedges of the CLC reflection band as well as one of the photonic defect modes in dual-mode lasing. Thanks to the optically bistable nature of CLC, such a white-light laser device can operate in quite an energy-saving fashion. This technique has potential to fulfill the present mainstream in the coherent white-light source.

Laser, known as the most unique light source with a distinctively long coherence length, has revolutionized several industries such as machinery, communications, and entertainment. The basis of laser emission contains the stimulating source and the gain media. Various solid and gas materials have been developed as laser gain media to generate lasers operating at different wavelengths. Recently, multi-color or multi-wavelength lasers emitted simultaneously from a single active material have been a subject of great interest. The realization of white lasers are the ultimate goal of laser scientists. Indeed, lasers that span the visible spectrum, particularly the red (R), green (G) and blue (B) colors, are useful for lighting^1^,^2^, color laser imaging and display^3^,^4^, biosensing^5^ as well as wavelength-division multiplexing^6^. As an illumination technology, lasers offer higher energy efficiencies and higher output powers than white light-emitting diodes (LEDs) and other traditional illuminants. Along this line, an organic white laser source was successfully demonstrated^7^. Most recently, an inorganic semiconductor laser source has also been developed with a monolithic multi-segment semiconductor nanostructure based on a quaternary alloy of ZnCdSSe enabling simultaneously lasing in the R, G and B colors^8^.

Regarded as soft photonic crystals (PCs), cholesteric liquid crystals (CLCs) have been manifested as effective lasing media because of the Bragg reflection (BR) stemming from their self-assembled, periodically helical structure^9^,^10^. The BR of a CLC affects a specifically, circularly polarized component of impinging light whose chirality is the same as that of the CLC. This causes the group velocity of the incident light to approach to zero at either reflection bandedge and, in turn, gives rise to a very high photonic density of states with a long dwell time, thereby sustaining the stimulated emission^11^. Apart from photonic bandedge lasing, intentionally placing a defect in a solid PC would possibly induce another mode of lasing, known as photonic defect-mode lasing. Defect-mode lasing is also accomplished by the long photon dwell time in the defect, giving a low-threshold laser mode^12^. In this sense, the interplay between soft CLC and solid PC in a hybrid structure has aroused a great interest of many research groups^13^,^14^ and such organo–inorganic hybrids for white laser sources have yet to be explored. In some reports, polymeric CLC films employed as PCs are incorporated with a dye-doped nematic liquid crystal (DDNLC) between them to form hybrid structures for simultaneous lasing^7^,^15^,^16^. Simultaneous R, G, and B lasing emissions are also proposed in the same structures^7^. The wavelengths of the RGB lasing emissions from the Fabry–Pérot cavity can be modulated electrically since the DDNLC acts as a tunable waveplate^16^. Unlike these organic devices, the “coupled” PC (CPC) as the focus of this study is a consolidation of a dye-doped CLC (DDCLC) sandwiched by a pair of unconventional dielectric mirrors serving as imperfect, one-dimensional (1-D) PCs. The BR band of the organic PC and the photonic bandgap (PBG) of the two identical, inorganic PCs are said to be “coupled” but allocated to different spectral regions, contrast to the single-mode lasing^17^. In this artificial composition of laser gain materials, both the defect-mode and the bandedge-mode lasing exist simultaneously, termed as the dual-mode lasing. Two of the emissions verge on the both edges of the BR band and the other one originates from the spiky transmission of defect mode in the spectral PBG.

Although the defect-mode lasing happens only as the excitation intensity going beyond the threshold, it is arduous to excite only a single mode within the broad PBG. Having the photonic density of states high enough to emit the laser, the central region of a broad PBG crowded with numerous defect-mode peaks inevitably covers multiple emissions induced by a pumping light with a reasonable power level. Given that a low-power pumping light leads to a limited output, differentiating the sensitivities between the one around the center and the others is necessary. Simply reducing the defect layer thickness within the PCs seems to be a good option, but it is, unfortunately, scarcely applicable to the device herein in that the emission efficiency of CLC lasing is strongly dependent of its thickness. As such, the only solution is to contract the width of the PBG. To consider a stricture upon the PBG, one might think of alternating the dielectric layers with a smaller refractive-index difference while increasing the number of the layers to several tens to reach an adequate reflectance. Another much more feasible solution adopted in this study is to implement a period-disturbing layer in each multilayer by which a single defect-mode peak divides the PBG into two parts in the transmission spectrum. Based on this CPC, a discrete trichromatic laser device covering the three primary colors was demonstrated as a facile white-light source. Furthermore, the electrically tunable CLC is bistable in the planar state and the focal conic state^18^,^19^.

## Results

**Figure 1:**
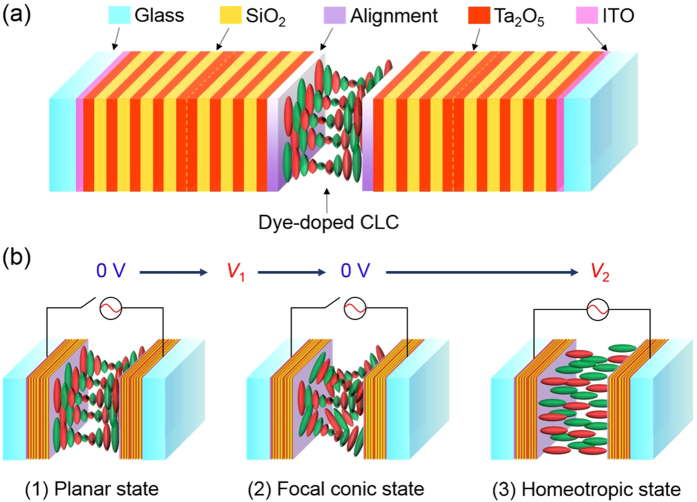
Schematics of (**a**) the CPC and (**b**) the corresponding configurations of the three CLC states. The red and green ellipsoids represent the dye and the LC molecules, respectively.

A detailed illustration about the structure of the CPC is shown in Fig. 1(a). The stacking for the structure can be coded as [GI(HL)4HH(LH)3]−P(D)P−[(HL)3HH(LH)4IG], where G is the glass substrate; I is the ITO electrode; H and L refer to the dielectrics of high and low refractive indices, respectively; P stands for the polyimide alignment layer; D represents the DDCLC composed primarily of the nematic host E44 and the chiral dopant R5011. The dielectric materials constituting the 1-D PCs are Ta_2_O_5_ and SiO_2_ whose respective refractive indices *n*_H_ = 2.18 and *n*_L_ = 1.47 at the wavelength of 550 nm. Two types of dielectric PCs, designated PC^R^ and PC^B^, were designed to render their PBGs centered at 640 and 450 nm, respectively. CLCs, as have been known, exhibit at least two optically stable states (at null voltage)—planar and focal conic states. The manipulations (or switching) and the configurations of the molecular orientations in three states are depicted in Fig. 1(b). The planar state is the initial state, and an intermediate voltage *V*_1_ leads to the focal conic state where the transparency gets low because of scattering. The homeotropic state can only be sustained with large voltage *V*_2_, typically larger than 50 V for a few-micron-thick CLC cell.

**Figure 2:**
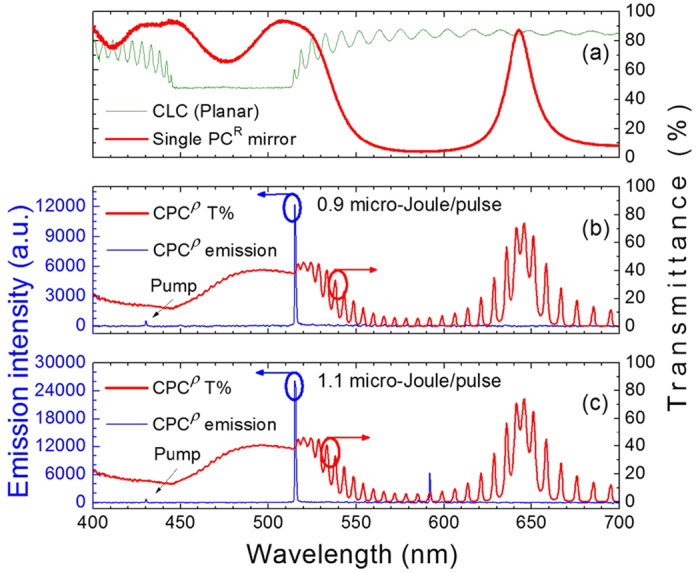
Transmittance and emission spectra of CLC (green, top chart), PC^R^ (red, top chart) and CPC^ρ^ composed of a 15-*μ*m-thick CLC in the planar state (middle and bottom charts). The blue curves are stimulated emission along with the pumping light at around 430 nm.

The transmission spectra of a 15-*μ*m-thick CLC cell and a single PC^R^ substrate are displayed in Fig. 2(a). The broad PBG is divided by a defect-mode peak at the PBG center around 640 nm, and the CLC BR is located right adjacent to the left half PBG. Combining a sandwiched DDCLC and a pair of PC^R^ substrates, we fabricated the assembly CPC^ρ^. Fluorescent dyes (C540A and LD688) as the primitive light producers were introduced to absorb the pumping energy, thereby generating a few spectral fluoresce humps that match the designated lasing modes. An Nd:YAG laser with an optical parameter oscillator allows the pumping light to be tuned between 420 and 680 nm; it was attuned at around 430 nm for CPC^ρ^ to meet the maximum absorption of the fluorescent dyes. The whole experimental setup is illustrated in Supplementary Fig. S1.

**Figure 3:**
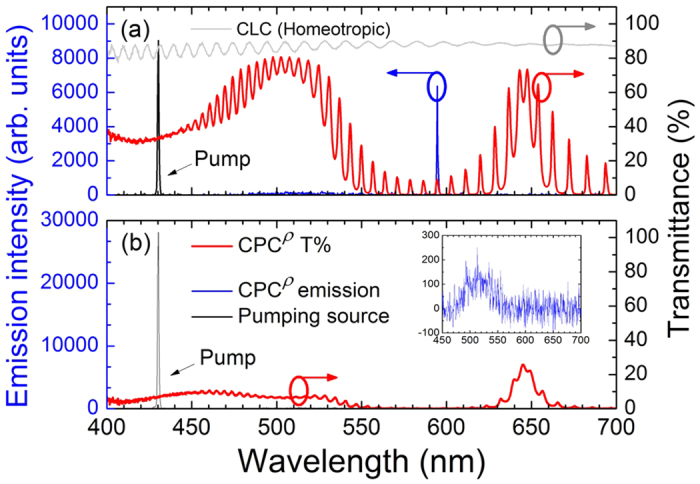
Transmittance and emission spectra of CPC^ρ^ in (**a**) the homeotropic state and (**b**) the focal conic state. Inset in (**b**), expanded scale at low emission intensities in the same state.

In general, the defect-mode lasing has lower threshold than does the bandedge-mode lasing^20^. As can be seen in Fig. 2(b), however, we succeeded in permitting bandedge lasing (at wavelength of 515.17 nm) alone to be stimulated. This is because the intensity distribution of the dye fluorescence was intentionally manipulated, by blending two dyes at an appropriate concentration ratio (see Supplementary Fig. S2). Otherwise, the defect-mode lasing would always be induced before the bandedge lasing, forbidding the bandedge mode to emit solely in the CPC. Note that defect-mode lasing alone can occur when a voltage *V*_2_ (>52.5 V, specifically) is applied to unwind the CLC, changing the state to the homeotropic state (Fig. 1(b)) and, in turn, eliminating the BR. Based on this particular design, the lasing can, therefore, be electrically switched among the bandedge-mode (for lasing at 515.17 nm, Fig. 2(b)), the dual-mode (lasing at both 515.17 and 591.80 nm, Fig. 2(c)), and the defect-mode (lasing at 594.53 nm, Fig. 3(a)) emissions. The optical properties in the homeotropic state are similar to those in the case of filling nematic LC as the defect layer^21^. Between the planar state and homeotropic state is the focal conic state, in which randomly arranged double-cones forming multiple domains strongly scatter the excitation light. This configuration is optically stable, meaning that the state, once formed, remains even after the voltage is released. Figure 3(b) was acquired in such stable light-scattering state, where the inset isolates the emission spectrum from the large-scale signals. Here very little fluorescence was collected; there was no laser at all because the emission was not stimulated in the first place. Intuitively, bandedge-mode lasing does not exist as the focal conic state has no BR. The stimulated emission in DDCLCs requires fluorescent dyes to transfer the pumping light into some other wavelengths that can well be amplified by the reflection. The scattering kept the dyes from absorbing the incident light effectively, therefore the remaining weak fluorescence could not exceed the threshold for defect-mode lasing. This connotes that a unidirectional orientation of the CLC helical axis is a prerequisite for the laser excitation in DDCLCs^22^.

**Figure 4:**
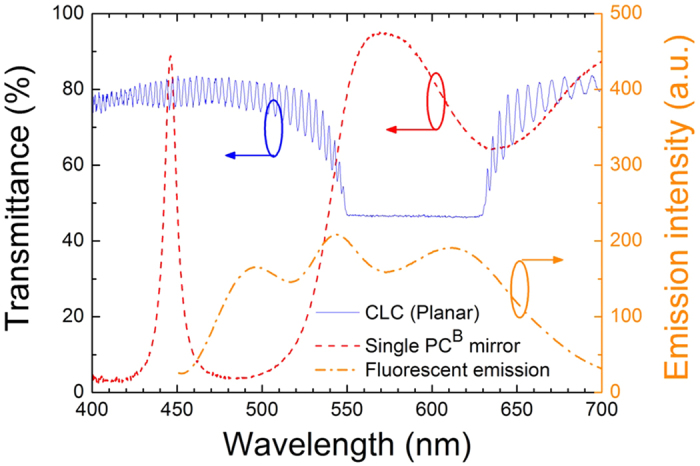
Spectra of individual components forming CPC^β^. All three spectra were measured separately.

**Figure 5:**
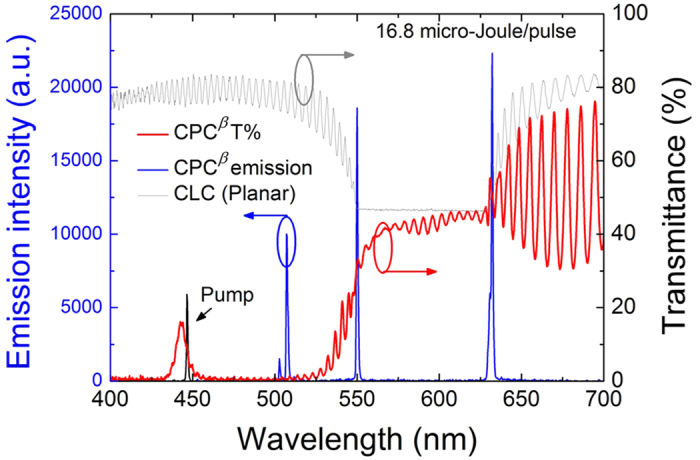
The white-light lasing spectrum and the referencing transmittance spectra of CPC^β^ and the CLC alone in the planar state.

In the CPC^ρ^ structure mentioned above, the high-frequency bandedge of the BR near 445 nm is located in the absorption band of the dye mixture. As such, there lacked blue emission to be observed. The CPC^β^, comprising a ~15-*μ*m-thick DDCLC sandwiched by two PC^B^ substrates, was elaborated to fix this problem and, meanwhile, to demonstrate the other type of CPCs. The two bandedges of the BR in this design was allocated at around 550 nm and 630 nm whereas the PBG of the PC^B^ took the seat next to the BR as shown in Fig. 4. The dye composition (C540A, PM580 and LD688) in CPC^β^ was carefully adjusted so as to fluoresce in three lasing emissions roughly representing the primary colors. The fluorescence spectrum is illustrated in Fig. 4 and the absorption spectrum can be found in Supplementary Fig. S3. The artificial defect-mode peak around 446 nm, emanating from the central Ta_2_O_5_ layer in double thickness in the dielectric multilayer, allowed the pumping light to penetrate the PC^B^ substrate for dye absorption to stimulate the emissions. With all these endeavors, a discrete white-light laser beam composed of three quasi-primary colors can be achieved. Figure 5 displays the emission and transmittance spectra of the CPC^β^ in the planar state as well as the transmittance spectrum of the CLC alone. One can see that the fluorescence intensities associated with the BR bandedges were not significantly higher than that of the defect-mode lasing intensity, presumably due to some part of the blue emission being re-absorbed by the dyes. The disparity between the two at the verges of the BR is attributed to the standing waves in the cavity having preferred polarization which benefits the long-wavelength side^23^. Here the defect-mode laser was supposed to contribute to the shorter-wavelength color but is hardly referred to as blue as it pinned 507 nm (bluish green). The other two, nevertheless, scored the prototypical positions of red and green at 632 nm and 550 nm, respectively. A preferred blue emission, say, at 450 nm, from CPC^β^ is enslaved to the dye absorption and illumination spectra; it can be generated only if the dye is able to absorb pumping light of a wavelength of the shorter wavelength to permit vanished absorption at the preferred emission. Poring over the emission spectrum (Fig. 5) one might notice a satellite peak (at 502 nm) on the left shoulder of the “blue” peak, suggesting that the control of the single defect-mode emission was not as good as in CPC^ρ^. The reason is that in this particular CPC the thickness of the mesogenic defect layer; i.e., the cell gap, was somewhat larger due to the limited sample fabrication accuracy. The defect-mode peak density per frequency can be extracted from the transmittance spectra (Supplementary Tables S1 and S2); the density of 0.162 THz^–1^ in CPC^β^ is 11% denser compared with that of 0.146 THz^–1^ in CPC^ρ^. The quantitative relations among the pumping energy, the defect-mode density and the width of PBG remain to be explored. As same as in the CPC^ρ^, the focal conic state and the homeotropic state were also attainable through electrical switching (as shown in Supplementary Fig. S4).

**Figure 6:**
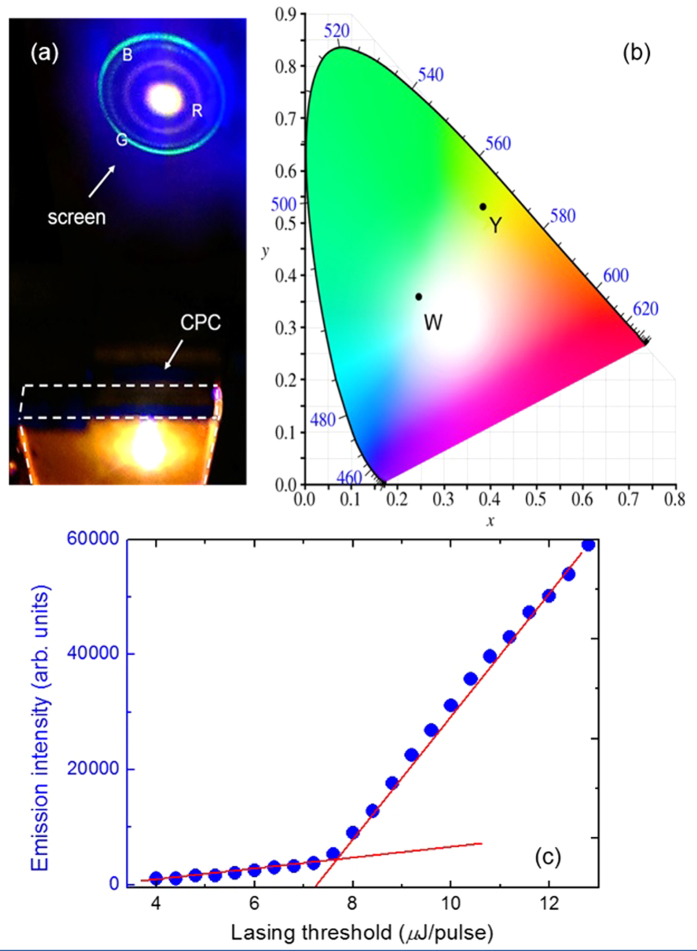
(**a**) Photograph of a tricolor CPC laser device and its discrete emissions on a screen, (**b**) the true color space coordinates of the laser on the CIE 1931 chromaticity diagram, and (**c**) the pumping energy-dependent intensity of the lasing emitted from the CPC^β^ device.

A genuine photo of the CPC^β^ as a laser device is shown in Fig. 6(a), accompanied by the CIE1931 color space chromaticity diagram indicating the color of the discrete white-light laser as shown in Fig. 6(b). The color space data were calculated based on the emission spectrum as shown in Fig. 5. The Y point, with a coordinate of (*x, y*) = (0.3831, 0.5308), corresponds to the primitive color mixing of the three stimulated emissions. On the other hand, the W point (*x* = 0.2459; *y* = 0.3637) accretes the additionally scattered intensity of the pumping light (i.e., mixing of four colors at wavelengths of 446, 507, 550 and 632 nm). In the photo, the actual lasing color is supposed to appear flaxen. Here the white appearance of the CPC laser spot is contributed by the scattered pumping light as the ubiquitous blue background. The sharp refraction rings of the three colors on the screen imply that the light coherence of the stimulated lasing was tangible. Figure 6(c) depicts the lasing intensity of CPC^β^ as a function of the pumping energy. A threshold can be deduced at about 7.4 µJ/pulse, beyond which the emission intensity drastically increased. Here the somewhat larger threshold is attributable to the required pumping energy to stimulate the blue-color component. Note that the lasing threshold was measured from the experimental system, where an Nd:YAG pulsed laser was used to excite the DDCLC cells. The pumping energy of the incident pulses was adjusted through the combination of a half-wave plate and a linear polarizer to control the incident pulsed energy. The lasing output from the CPC cell was received by a fiber-based spectrometer in the direction of the cell normal (see Fig. S1). A plot of linewidth (FWHM) vs. lasing threshold shows a typical behavior of CLC lasing, with a minimal FWHM of 1.7 nm at the lasing threshold of 8.4 *μ*J/pulse (data not shown). The linewidth of each lasing peak is *ca.* 2–3 nm throughout this study.

## Discussion

To the best of our knowledge, this is the first demonstration of a dual-mode laser source being able to simultaneously yield three colors of stimulated light. Although the discrete white-light source revealed herein does not render impeccable white light, the concept is firmly explicit and the performance could be optimized by choosing suitable dyes and fine tuning the CPC structure. The organo–inorganic CPC can not only generate three lasers that cover the quasi-primary colors with a single pump but also be electrically switched among the defect-mode, bandedge-mode and dual-mode lasing. With such switchability, lasing wavelength can be altered back and forth in a wavelength range beyond 100 nm and in a comparatively short time as compared with previous works^24^,^25^,^26^,^27^. As for the semiconductor white-light laser which is based on multi-segment nanostructure, CPC takes the advantages of cost effectivity, facile fabrication and color tunability in a single cell of device. What is more attractive might be that the CPC can be pumped using a CW laser, thereby generating a de-facto non-staccato single-color, two-color, three-color or white-light laser for display, lighting and other applications. By integrating a compact diode laser as pumping source^28^, a size-reduced system can be achieved to make the proposed applications more feasible.

## Methods

In PC^R^ the layer thicknesses of H (Ta_2_O_5_) and L (SiO_2_) are 73.4 and 108.8 nm and in PC^B^ they are 51.6 and 76.5 nm, respectively. Such designs ensure that the optical path length, namely, the product of the refractive index and the thickness, is identical for each dielectric layer and that the optical length is a quarter of the central wavelength of the PBG. The polyimide (PI) used as alignment layers is SE–150 from Nissan Chemical Co., which was spin-coated atop a PC multilayer on a glass substrate. Each CPC was fabricated with two identical PI-coated multilayer substrates, separated by rod-shape spacers of the diameter of 15 *μ*m. Because of the opposite rubbing directions imposed on the alignment layers, the LC as a filler took an anti- parallel (180°) orientation on the surfaces.

The DDCLC mixture in CPC^ρ^ contained a nematic LC (E44 from Daily-Polymer Co.) as the host and 3.23-wt% chiral dopant R5011 (from Jiangsu Hecheng Advanced Materials Co.), forming a right-handed CLC. It also comprised two fluorescent dyes: 0.70-wt% Coumarin 540A and 0.04-wt% LD 688 (both from Exciton). The anisotropic refractive indices of E44 are *n*_e_ = 1.790 and *n*_o_ = 1.548 at room temperature. In CPC^β^, the composition of the DDCLC is E44: R5011: Coumarin 540A: LD 688: Pyrromethene 580 (also from Exciton) = 96.62: 2.55: 0.7: 0.06: 0.07 wt%. Prior to the introduction into an empty cell formed by a pair of PI-coated PC^R^ or PC^B^ substrates, both of the DDCLC mxitures were individually and homogeneously concocted on a hot plate to promote uniformity. A DDCLC cell was then prepared by filling the cell with a DDCLC mixture by capillary action, giving a uniform film of ~15 *μ*m in thickness sandwiched between two PCs and, thus, forming a desired CPC.

## Additional Information

**How to cite this article**: Huang, J.-C. *et al.* Electrically switchable organo–inorganic hybrid for a white-light laser source. *Sci. Rep.* **6**, 28363; doi: 10.1038/srep28363 (2016).

## Supplementary Material

Supplementary Information

## References

[b1] Neumann A. *et al.* Four-color laser white illuminant demonstrating high color-rendering quality. Opt. Express 19, 982–990 (2011). 10.1364/OE.19.00A982 21747570

[b2] Wierer J. J., Tsao J. Y. & Sizov D. S. Comparison between blue lasers and light emitting diodes for future solid-state lighting. Laser Photon Rev. 7, 963–993 (2013).

[b3] Zhao J., Jiang H. & Di J. Recording and reconstruction of a color holographic image by using digital lensless Fourier transform holography. Opt. Express 16, 2514–2519 (2008). 18542331 10.1364/oe.16.002514

[b4] Chellappan K. V., Erden E. & Urey H. Laser-based displays: a review. Appl. Opt. 49, 79–98 (2010). 10.1364/AO.49.000F79 20820205

[b5] Kotani A. *et al.* EndoV/DNA ligase mutation scanning assay using microchip capillary electrophoresis and dual-color laser-induced fluorescence detection. Anal. Methods 4, 58–64 (2012).

[b6] Cossu G., Khalid A. M., Choudhury P., Corsini R. & Ciaramella E. 3.4 Gbit/s visible optical wireless transmission based on RGB LED. Opt. Express 20, 501–506 (2012). 10.1364/OE.20.00B501 23262894

[b7] Ha N. Y. *et al.* Simultaneous red, green, and blue lasing emissions in a single pitched cholesteric liquid crystal system. Adv. Mater. 20, 2503–2507 (2008). 10.1038/nmat2045 17994028

[b8] Fan F., Turkdogan S., Liu Z., Shelhammer D. & Ning C. Z. A monolithic white laser. Nat. Nanotechnol. 10, 796–803 (2015). 26214252 10.1038/nnano.2015.149

[b9] Goldberg L. & Schnur J. Tunable internal-feedback liquid crystal-dye laser. US Patent No. 3, 771, 065 (1973).

[b10] Ilchishin I. P., Tikhonov E. A., Tishchenko V. G. & Shpak M. T. Generation of a tunable radiation by impurity cholesteric liquid crystals. JETP Lett. 32, 24–27 (1980).

[b11] Kopp V. I., Fan B., Vithana H. K. M. & Genack A. Z. Low-threshold lasing at the edge of a photonic stop band in cholesteric liquid crystals. Opt. Lett. 23, 1707–1709 (1998). 18091891 10.1364/ol.23.001707

[b12] Yablonovitch E. Inhibited spontaneous emission in solid-state physics and electronics. Phys. Rev. Lett. 58, 2059–2062 (1987). 10034639 10.1103/PhysRevLett.58.2059

[b13] Matsuhisa Y., Ozaki R., Yoshino K. & Ozaki M. High Q defect mode and laser action in one-dimensional hybrid photonic crystal containing cholesteric liquid crystal. Appl. Phys. Lett. 89, 101109–1–3 (2006).

[b14] Matsuhisa Y. *et al.* Cholesteric liquid crystal laser in a dielectric mirror cavity upon band-edge excitation. Opt. Express 15, 616–622 (2007). 19532283 10.1364/oe.15.000616

[b15] Song M. H. *et al.* Defect-mode lasing with lowered threshold in a three-layered hetero-cholesteric liquid-crystal structure. Adv. Mater. 18, 193–197 (2006).

[b16] Choi H. *et al.* Broadband cavity-mode lasing from dye-doped nematic liquid crystals sandwiched by broadband cholesteric liquid crystal bragg reflectors. Adv. Mater. 22, 2680–2684 (2010). 20432476 10.1002/adma.200904110

[b17] Matsuhisa Y., Ozaki R., Ozaki M. & Yoshino K. Single-mode lasing in one-dimensional periodic structure containing helical structure as a defect. Jpn. J. Appl. Phys. 44, 629−632 (2005).

[b18] Hsiao Y.-C., Wu C.-Y., Chen C.-H., Zyryanov V. Y. & Lee W. Electro-optical device based on photonic structure with a dual-frequency cholesteric liquid crystal. Opt. Lett. 36, 2632–2634 (2011). 21765491 10.1364/OL.36.002632

[b19] Hsiao Y.-C., Tang C.-Y. & Lee W. Fast-switching bistable cholesteric intensity modulator. Opt. Express 19, 9744–9749 (2011). 21643231 10.1364/OE.19.009744

[b20] Jeong S. M. *et al.* Defect mode lasing from a double-layered dye-doped polymeric cholesteric liquid crystal films with a thin rubbed defect layer. Appl. Phys. Lett. 90, 261108–1–3 (2007).

[b21] Ozaki R., Matsui T., Ozaki M. & Yoshino K. Electro-tunable defect mode in one-dimensional periodic structure containing nematic liquid crystal as a defect layer. Jpn. J. Appl. Phys. 41, 1482–1484 (2002).

[b22] Furumi S., Yokoyama S., Otomo A. & Mashiko S. Electrical control of the structure and lasing in chiral photonic band-gap liquid crystals. Appl. Phys. Lett. 82, 16–18 (2003).

[b23] Schmidtke J., Stille W., Finkelmann H. & Kim S. T. Laser emission in a dye doped cholesteric polymer network. Adv. Mater. 14, 746–749 (2002).

[b24] Wang H.-T., Lin J.-D., Lee C.-R. & Lee W. Ultralow-threshold single-mode lasing based on a one-dimensional asymmetric photonic bandgap structure with liquid crystal as a defect layer. Opt. Lett. 39, 3516–3519 (2014). 24978525 10.1364/OL.39.003516

[b25] Funamoto K., Ozaki M. & Yoshino K. Discontinuous shift of lasing wavelength with temperature in cholesteric liquid crystal. Jpn. J. Appl. Phys. 42, 1523–1525 (2003).

[b26] Chanishvili A. *et al.* Phototunable lasing in dye-doped cholesteric liquid crystals. Appl. Phys. Lett. 83, 5353–5355 (2003).

[b27] Chanishvili A. *et al.* Widely tunable ultraviolet-visible liquid crystal laser. Appl. Phys. Lett. 86, 051107–1–3 (2005).

[b28] Chi Y.-C. *et al.* Phosphorous diffuser diverged blue laser diode for indoor lighting and communication. Sci. Rep. 5, 18690 (2015). 26687289 10.1038/srep18690 PMC4995634

