# Supplementary material for: Electrically switchable organo–inorganic hybrid for a white-light laser source
Source: Sci Rep. 2016 Jun 21;6:28363. doi: 10.1038/srep28363 (PMC4914921; doi:10.1038/srep28363)
Supplement: Supplementary Information [file srep28363-s1.pdf]

Supporting Information

Electrically switchable organo–inorganic hybrid for a white-light laser source

Jui-Chieh Huang[1,†], Yu-Cheng Hsiao[2,†], Yu-Ting Lin[2], Chia-Rong Lee[3] & Wei Lee[2,*]

[1] Institute of Photonic System, College of Photonics, National Chiao Tung University, Guiren Dist., Tainan 71150, Taiwan.
[2] Institute of Imaging and Biomedical Photonics, College of Photonics, National Chiao Tung University, Guiren Dist., Tainan 71150, Taiwan.
[3] Department of Photonics, National Cheng Kung University, Tainan 70101, Taiwan.
[†]These authors contributed equally to this work.
[*]Correspondence and requests for materials should be addressed to W.L. (email: wlee@nctu.edu.tw).

This document provides supplementary information to “Electrically switchable organo–inorganic hybrid for a white-light laser source”. Details from material properties, device fabrication procedures to experiment setups and addition data including photos are provided herein below.

The transmittance spectra were measured with a fiber-optic spectrometer (Ocean Optics, HR2000+) whereas the absorption and fluorescence spectra were acquired with a monochromator (Princeton Instruments, Acton SP2150). The experimental setup is schematically illustrated in Fig. S1. The pumping source is a Q-switched Nd:YAG third-harmonic generation pulse laser (wavelength: 355 nm, Spectra Physics, Quanta-Ray Lab- 130) with a pulse duration of 8 ns and a repetition rate of 10 Hz. The optical parametric oscillator (GWU OPO) tuned the input beam into a different laser wavelength anywhere between 400 and 700 nm. The power of the pumping light impinging on the CPC was controlled by an aperture. The single pump laser was focused with a lens of 5-cm-long focal length onto the CPC sample at a ~20° incident angle to discern the pumping beam and the stimulated emissions when collecting data. The emission spectra were obtained with an optical fiber coupled to an Ocean Optics spectrometer model Jaz-Combo-2 with 0.35-nm resolution. A function generator (Picotest, G5100a) provided square-wave

voltage of 1-kHz, amplified by a voltage amplifier (FLC electronics, A400) by 20× before applied across the CPC.

The absorption and the fluorescence spectra of the dye mixture in CPC$^{\rho}$ are illustrated in Fig. S2 and the individual and the coalesced absorption spectra of the three dyes employed in CPC$^{\beta}$ are shown in Fig. S3. In Fig. S2, the spectra of the two components of the dye mixture are not individually displayed. Here the dye absorption was measured from the nematic LC host without the chiral dopant. The concentrations of the dyes for the absorption measurement were same as for the DDCLC. Figures S4 and S5 depict the spectra of CPC$^{\beta}$ in the focal conic state at null voltage and the homeotropic state sustained by a 55-V applied voltage, respectively.

To quantify the density of the defect modes, we first identified the positions of the peaks with the Lorentzian peak fitting, then transformed the wavelengths into frequencies via the equation $c = f\lambda$ since the defect modes are supposed to be distributed symmetrically at the both sides of the PBG in the frequency domain. Taking the intervals of the defect-mode positions in frequency, we then have the average interval $\bar{i}$ between defect- mode peaks. The reciprocal of $\bar{i}$ means how many defect modes in a frequency range, per THz used herein, which represents the defect-mode density. The defect-mode positions in both wavelength and frequency as well as the frequency intervals are listed in Table S1 for CPC$^{\rho}$ and in Table S2 for CPC$^{\beta}$. Since two adjacent defect-mode frequencies produce one interval, it behooves one datum short in both tables. The peaks were identified as the CPCs were in the homeotropic state (from Fig. 3 in the main document and from Fig. S5) to avoid any perturbation from the CLC with different pitches. In Table S1, those defect modes located on the artificial peak (which divides the PBG into two) had smaller intervals, were excluded from the $\bar{i}$ calculation. Data in Table S2 have no such

concern because the small peaks on the designated peak were too subtle to identify, and therefore only peaks on the right half of the PBG were located in Table S2. The standard deviation of the defect-mode intervals in CPC^{ρ} is 0.11 THz, reflecting +0.0022 and −0.0023 THz^{-1}; in CPC^{β} the numbers are +0.0059 and −0.0069 THz^{-1}.

Figure S6 presents four photos of CPC^{β}. By tuning pumping intensity lower than the defect-mode threshold, the bottom two photos show the lasing color of the bandedge mode only. The bright spot to the right of the CPC lasing in each photo is the pumping light.

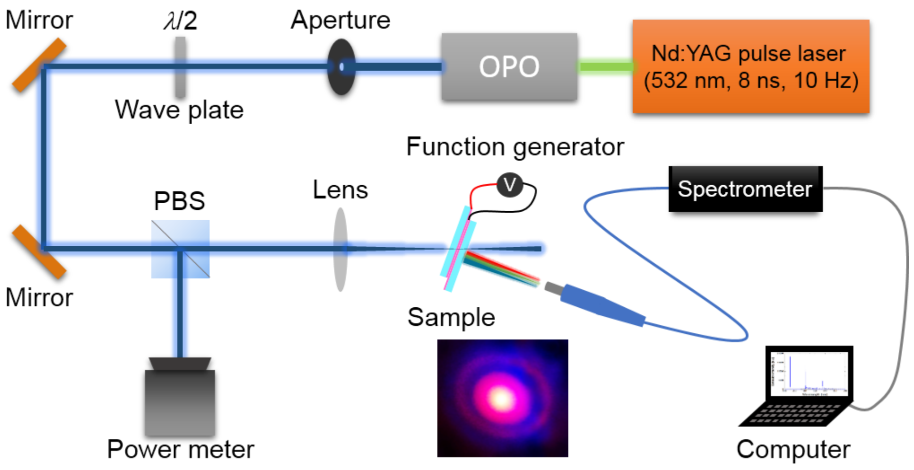

Figure S1. Schematic of the experimental setup. The tungsten halogen light source used in transmittance spectroscopy is not shown.

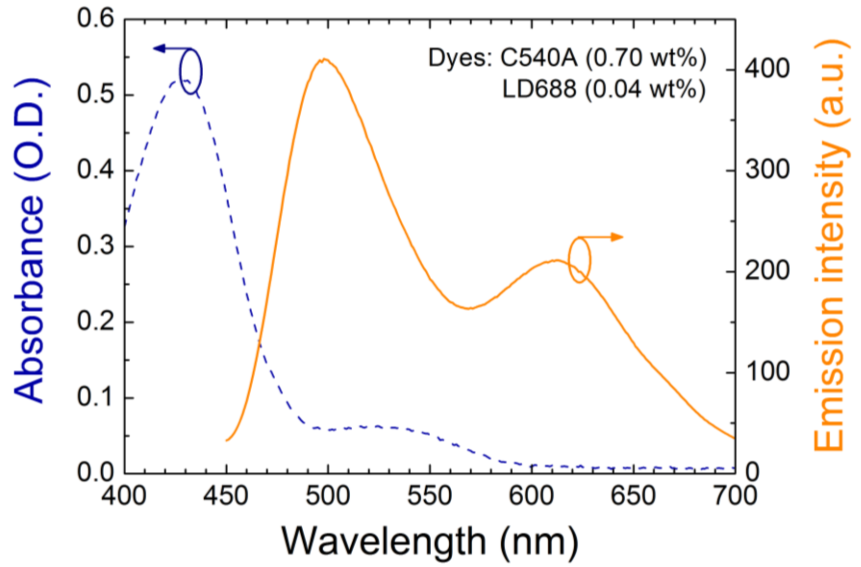

Figure S2. Absorption and the fluorescence spectra of the dye mixture used in CPC$^{\rho}$. The measurements were taken from a DDLC sample, without PC and chiral dopant.

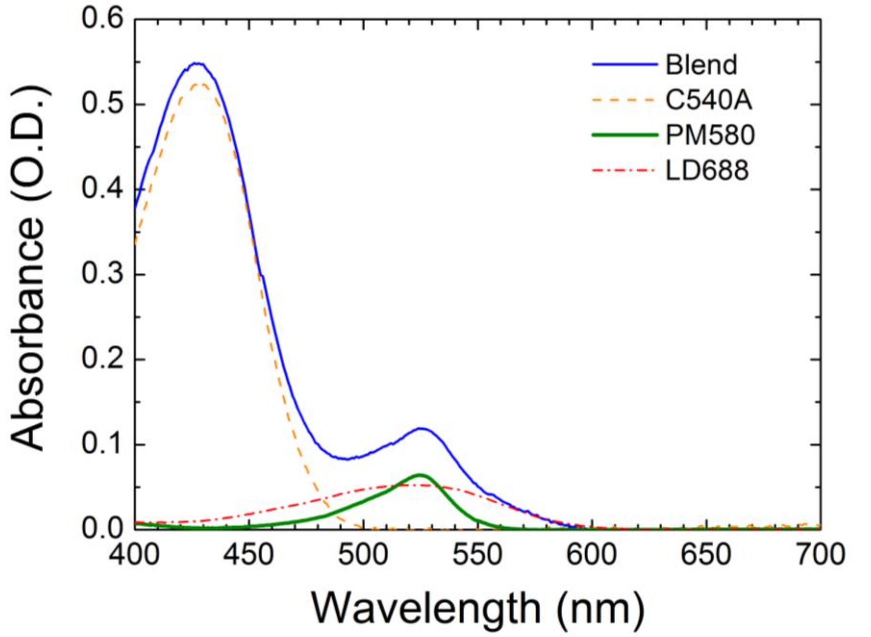

Figure S3. Absorbance spectra of the dyes used in CPC$^{\beta}$. The coalesced absorption in blue solid line corresponds to the absorbance of the three-component mixture.

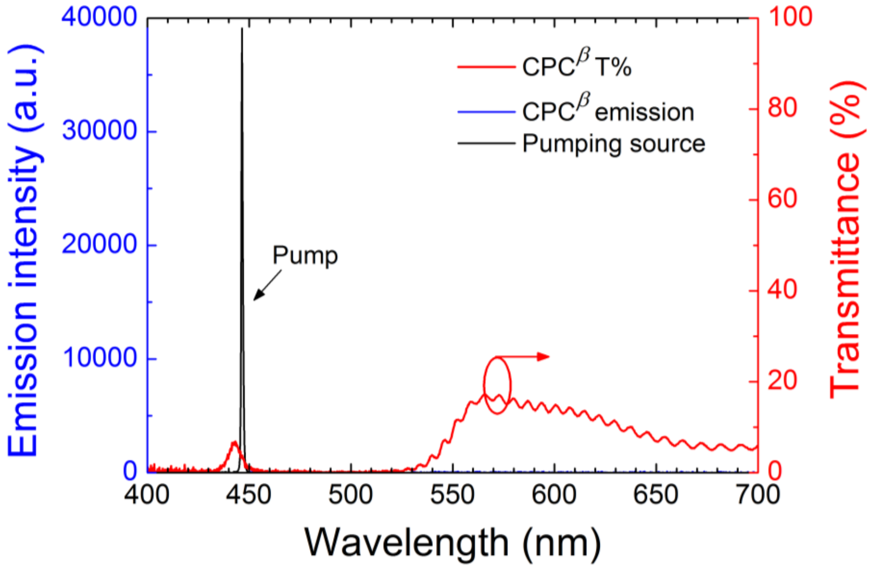

Figure S4. Emission and transmittance spectra of CPC$^{\beta}$ in focal conic state.

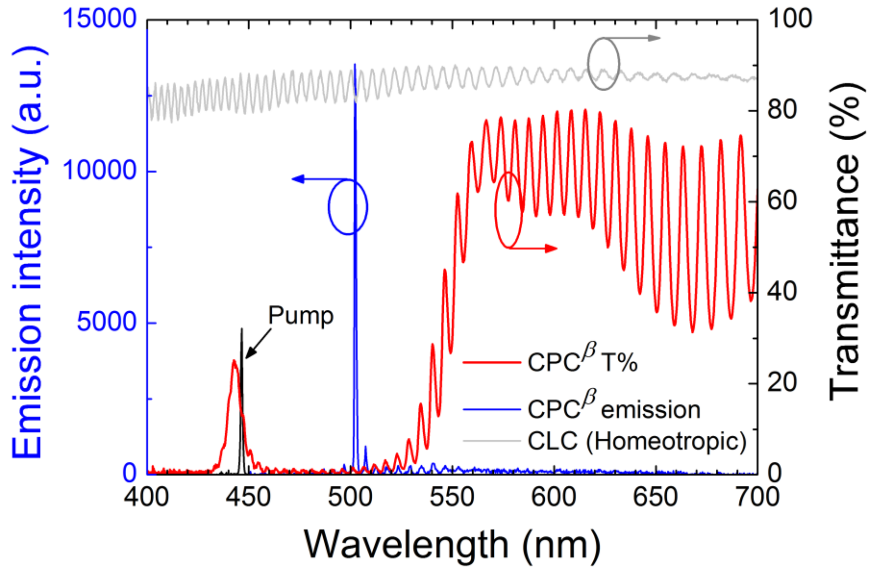

Figure S5. The emission and the transmittance spectra of CPC$^{\beta}$ in the homeotropic state sustained at 55 V.

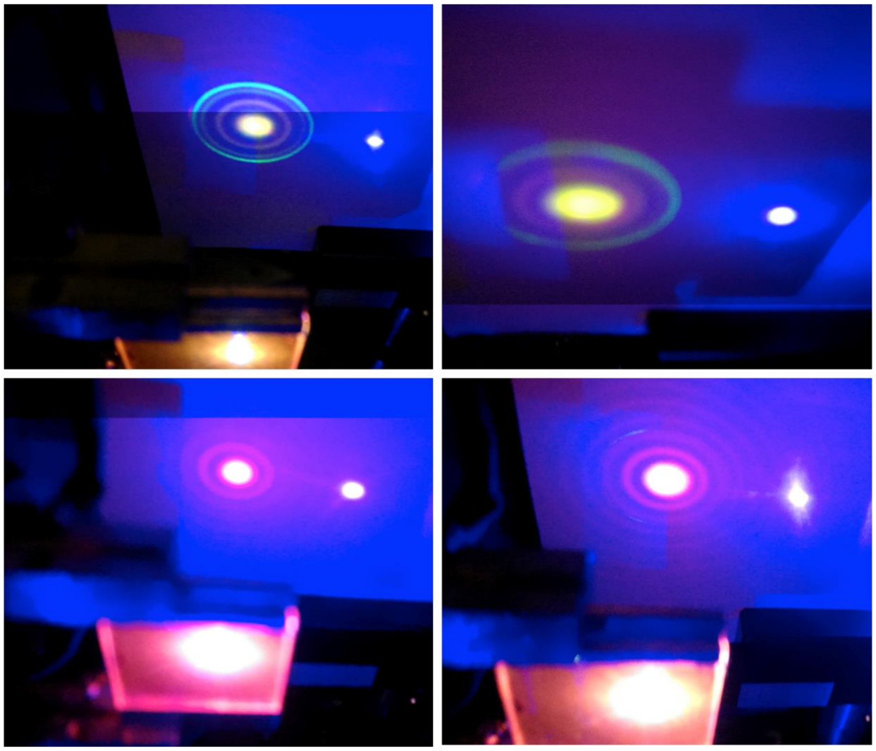

Figure S6. More lasing photos with different colors. All of these pictures were taken of the CPC$^{\beta}$. Only bandedge-mode lasing was stimulated in the bottom two.

Table S1. Defect-mode positions, intervals and the average defect-mode density of CPC$^{\rho}$. The defect modes around the artificial peak (located at the center of the broad PBG) in the shaded area are excluded from calculation of the mode density.

Position in λ (nm)	Position in f (THz)	Interval (THz)
549.8	545.3	6.71
556.6	538.6	6.74
563.7	531.8	6.85
571.0	525.0	6.85
578.6	518.1	6.77
586.3	511.4	7.02
594.4	504.4	6.91
602.7	497.4	6.89
611.1	490.6	6.86
619.8	483.7	6.67
628.5	477.0	6.14
636.6	470.9	4.72
643.1	466.2	3.19
647.5	463.0	4.49
653.9	458.5	5.93
662.4	452.6	6.53
672.1	446.0	6.84
682.6	439.2	7.01
693.7	432.2	
Average interval $\bar{\iota}$		6.84 THz^{-1}
Defect-mode density (reciprocal of $\bar{\iota}$)		0.146 THz^{-1}

Table S2. Defect-mode positions, intervals and the average defect-mode density of CPC^{β}.

Position in λ (nm)	Position in f (THz)	Interval (THz)
458.8	653.4	6.15
463.2	647.3	6.27
467.7	641.0	6.15
472.2	634.9	6.15
476.8	628.7	5.47
481.0	623.2	6.46
486.1	616.8	5.91
490.8	610.9	6.21
495.8	604.6	6.29
501.0	598.4	6.36
506.4	592.0	5.84
511.5	586.2	6.30
517.0	579.9	6.35
522.7	573.5	6.31
528.5	567.2	6.24
534.4	561.0	
Average interval $\bar{\imath}$		6.16 THz^{-1}
Defect-mode density (reciprocal of $\bar{\imath}$)		0.162 THz^{-1}
